# Methylprednisolone Induces Extracellular Trap Formation and Enhances Bactericidal Effect of Canine Neutrophils

**DOI:** 10.3390/ijms22147734

**Published:** 2021-07-20

**Authors:** Nicole Steffensen, Rabea Imker, Simon Lassnig, Marcus Fulde, Johanna C. Rieder, Nicole de Buhr

**Affiliations:** 1 Department of Small Animal Medicine and Surgery, University of Veterinary Medicine Hannover, Foundation, 30559 Hannover, Germany; Nicole.Steffensen@tiho-hannover.de; 2 Department of Biochemistry, University of Veterinary Medicine Hannover, Foundation, 30559 Hannover, Germany; Rabea.Imker@tiho-hannover.de (R.I.); simon.lassnig@tiho-hannover.de (S.L.); 3 Research Center for Emerging Infections and Zoonoses, University of Veterinary Medicine Hannover, Foundation, 30559 Hannover, Germany; 4 Centre for Infection Medicine, Institute of Microbiology and Epizootics, Freie Universität Berlin, 14163 Berlin, Germany; Marcus.Fulde@fu-berlin.de

**Keywords:** NETs, methylprednisolone, free DNA, dog, Gram negative bacteria, Gram positive bacteria

## Abstract

Methylprednisolone is a glucocorticoid and can negatively influence immune defense mechanisms. During bacterial infections in the dog, neutrophils infiltrate infected tissue and mediate antimicrobial effects with different mechanisms such as phagocytosis and neutrophil extracellular trap (NET) formation. Here, we investigated the influence of methylprednisolone on canine NET formation and neutrophil killing efficiency of Gram positive and Gram negative bacteria. Therefore, canine blood derived neutrophils were treated with different concentrations of methylprednisolone over time. The survival factor of *Staphylococcus pseudintermedius*, *Streptococcus canis* or *Escherichia coli* was determined in presence of stimulated neutrophils. Additionally, free DNA and nucleosomes as NET marker were analyzed in supernatants and neutrophils were assessed for NET formation by immunofluorescence microscopy. Methylprednisolone concentrations of 62.5 and 625 µg/mL enhanced the neutrophil killing of Gram positive bacteria, whereas no significant influence was detected for the Gram negative *Escherichia coli*. Interestingly, higher amounts of free DNA were detected under methylprednisolone stimulation in a concentration dependency and in the presence of *Streptococcus canis* and *Escherichia coli*. The nucleosome release by neutrophils is induced by bacterial infection and differs depending on the concentration of methylprednisolone. Furthermore, immunofluorescence microscopy analysis identified methylprednisolone at a concentration of 62.5 µg/mL as a NET inducer. In summary, methylprednisolone enhances NET-formation and time-dependent and concentration-dependent the bactericidal effect of canine neutrophils on Gram positive bacteria.

## 1. Introduction

The glucocorticoid methylprednisolone is used in veterinary medicine as an anti-inflammatory and immunosuppressive agent depending on the applied concentration [1,2,3]. The application is described in many immune-mediated diseases in the dog including immune-mediated hemolytic anemia and thrombocytopenia, chronic enteropathy, immune-mediated polyarthritis, meningoencephalomyelitis of unknown origin, canine atopic dermatitis and glomerulonephritis [1,4,5,6,7,8]. Administration of glucocorticoids result in stabilization of the cell membranes of granulocytes, mast cells and monocytes-macrophages. Furthermore, they inhibit phospholipase A2 and prevent the release of the pro-inflammatory cytokines Interleukin 1 (IL-1) and Interleukin 6 (IL-6). Additionally, a reduction in antigen processing and presentation by effects on macrophages and dendritic cells, direct suppression of the T cell function and reduced affinity of antibody to cell membrane epitopes is related to corticosteroid administration [1]. As an inflammatory reaction to a microbial infection, the innate immune system produces pro-inflammatory cytokines (IL-1, IL-6 and Tumor necrosis factor alpha (TNF-α)) and activates specific and nonspecific immune cells. Therefore, the use of corticosteroids can have negative effects on the host’s immune defense in cases of concurrent bacterial infections by inhibiting some immune-mediated pathways of inflammation [9,10].

Neutrophils are the first line of defense of the innate immune system of the host. They are recruited to the site of infection and can recognize invading microorganisms such as bacteria. Neutrophils can counteract invading bacteria by different mechanisms, including production of reactive oxygen species (ROS) and the release of antimicrobial peptides [11,12,13,14,15]. Furthermore, neutrophils can undergo phagocytosis and degranulation as well as exert antibacterial activity due to forming neutrophil extracellular traps (NETs) [16]. NETs are released as a reaction to different stimuli such as cytokines and pathogens. After stimulation, neutrophils release NETs consisting of a DNA backbone decorated with histones and granule-derived antimicrobial peptides and enzymes including neutrophil elastase, myeloperoxidase and proteinase. NETs can entrap, disarm and kill pathogens [12,14,16,17,18,19]. However, an overproduction or dysregulation of NETs can result in detrimental effects and are described in autoimmune diseases [20,21,22,23]. It is supposed that NETs have an impact on clot formation and immunothrombosis and are described in noninfectious diseases affecting the immune system such as immune-mediated hemolytic anemia in dogs [19,24,25,26]. In dogs, the NET release is described after parasitic infections with *Dirofilaria immitis*, *Trypanosoma cruzi* and *Toxoplasma gondii* [27,28,29]. In dogs suffering from a sepsis, NET formation was detected in different samples such as bronchoalveolar lavage and abdominal and pleural effusion [30]. Furthermore, NETs were detected in the endometrium of dogs with pyometra caused by *Escherichia coli* or *Streptococcus species* [31].

Several studies investigated the influence of drugs from natural as well as conventional medicine on neutrophil functions and bacterial killing. For example, Gum Arabic, which is a water-soluble polysaccharide with sugars used as a traditional medicine in Africa and India, has immunomodulatory effects on neutrophils by increasing the ROS production and phagocytic activity against *Escherichia coli* [32]. The breast cancer drug tamoxifen was described to enhance pro-inflammatory reactions of human neutrophils [33] and the antibiotic enrofloxacin enhances NET-formation [34]. Therefore, Munguia and Nizet discussed the importance of understanding the pharmacological activities of drugs on immune cells to identify hidden activities and to expand the antimicrobial armamentarium [35].

The aim of our study was to determine the effect of methylprednisolone, which is described as an anti-inflammatory and immunosuppressive drug, on canine neutrophils in cases of concurrent experimentally induced bacterial infections.

## 2. Results and Discussion

### 2.1. Methylprednisolone Enhances Neutrophil Killing of Gram Positve Bacteria, but Not of Gram Negative Bacteria

To analyze the influence of methylprednisolone on bacterial infections in dogs, we conducted neutrophil killing assays with *Staphylococcus* (*St.*) *pseudintermedius*, *Streptococcus* (*Sc.*) *canis* and *Escherichia* (*E.*) *coli* with and without methylprednisolone stimulation for a short period of incubation of one and three hours.

*St. pseudintermedius* is an opportunistic Gram positive pathogen and is part of the normal skin flora of most dogs. If the defense mechanisms of the host are reduced, skin lesions with the occurrence of a systemic inflammatory response syndrome are described [36,37,38]. Furthermore, *St. pseudintermedius* was found to be associated with urinary tract infections and respiratory tract disease [39,40,41]. *Sc. canis* is another commensal Gram positive bacterium found in the flora from skin and mucosa of dogs. However, an imbalance of the immune system can be associated with a toxic shock syndrome, necrotizing fasciitis, fibrinous bronchopneumonia and urinary tract infections triggered by *Sc. canis* [42,43,44,45,46,47]. The gut of the dog is colonized with *E. coli* as a normal commensal inhabitant starting directly after birth. A disturbance of the microbiome results in an imbalance of the normal intestinal flora. *E. coli* can cause gastrointestinal and extra-intestinal diseases such as urogenital tract infection in dogs [48,49,50,51,52].

**Figure 1:**
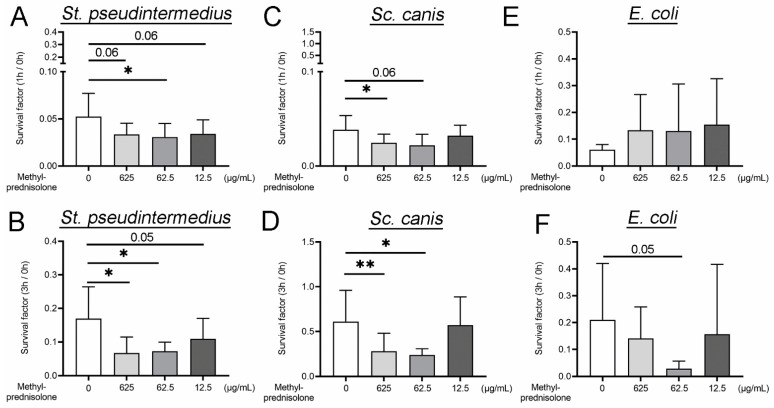
Methylprednisolone enhances neutrophil killing of Gram positive bacteria. The survival factor (SF) of bacteria were determined in the presence of methylprednisolone treated and untreated canine neutrophils. (**A**,**B**) SF of *St. pseudintermedius* was significantly decreased by 62.5 µg/mL methylprednisolone treatment after one hour of incubation (*n* = 4) and by 625 and 62.5 µg/mL methylprednisolone treatment after three hours of incubation (*n* = 5). (**C**,**D**) One hour after incubation, the SF of *Sc. canis* was significantly decreased by a methylprednisolone concentration of 625 µg/mL (*n* = 4). Methylprednisolone concentrations of 625 and 62.5 µg/mL decreased after three hours significantly the SF (*n* = 5). (**E**,**F**) The killing efficiency of neutrophils for *E. coli* was not significantly influenced by methylprednisolone over time at all (one hour: *n* = 4; and three hours: *n* = 5). Only after three hours of incubation, a statistical result with a *p* value of 0.05 was detected (*n* = 5). All graphs show the mean ± SD and statistical differences were detected by one-tailed paired Student’s *t*-test. Statistical results are presented for values lower than 0.1 and higher than 0.05 as an Arabic number and as the following: *, *p* < 0.05; **, *p* < 0.01.

The applied concentrations of methylprednisolone (625 µg/mL, 62.5 µg/mL and 12.5 µg/mL) were adapted to described plasma concentrations after systemic administration in the dog [53,54]. *St. pseudintermedius* showed after one and three hours of incubation a statistical lower survival factor (SF) at a methylprednisolone concentration of 62.5 µg/mL and after three hours incubation time additionally at 625 µg/mL compared to the SF of bacteria incubated with neutrophils alone (Figure 1A,B). The SF analysis of *Sc. canis* showed comparable results. A significant lower SF was detected after one hour of incubation with 625 µg/mL methylprednisolone and after three hours additionally at a concentration of 62.5 µg/mL (Figure 1C,D). Interestingly, the Gram negative bacteria *E. coli* was not significant enhanced killed by neutrophils stimulated with methylprednisolone (Figure 1E,F). These findings indicate a concentration-dependent influence of methylprednisolone on the bacterial killing of tested Gram positive bacteria in the presence of neutrophils, while no influence on the typically intracellularly killed bacteria *E. coli* was identified.

**Figure A1:**
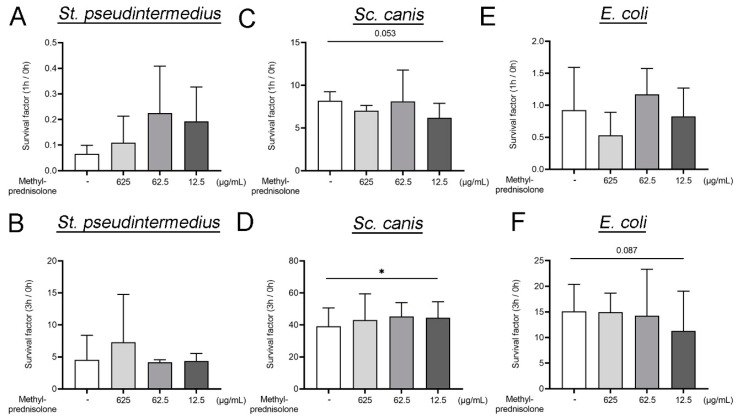
Gram positive and Gram negative bacteria can grow in the presence of methylprednisolone and canine plasma. The survival factor (SF) of bacteria were determined in the presence of different methylprednisolone concentrations, but without canine neutrophils as shown in Figure 1. (**A**,**B**) SF of *St. pseudintermedius* was not significantly influenced by methylprednisolone. (**C**,**D**) The highest SF of all analyzed bacteria was detected for *Sc. canis* in the presence of methylprednisolone (concentration 12.5 µg/mL). (**E**,**F**) The SF of *E. coli* was not significantly influenced by methylprednisolone over time at all. All graphs show the mean ± SD of three independent experiments with 10% plasma of three different donor dogs. Statistical differences were detected by one-tailed paired Student’s *t*-test calculated to the sample without methylprednisolone. Statistical results are presented for values lower than 0.1 and higher than 0.05 as Arabic numbers and as the following: *, *p* < 0.05.

In order to exclude a possible direct bactericidal effect of methylprednisolone on the investigated bacteria, the assay was repeated without canine neutrophils (Figure A1 in Appendix A). In the absence of neutrophils, all bacteria reached high survival factors. The highest survival factor was detected for *Sc. canis*. Furthermore, after three hours incubation no significant bactericidal effect by all tested concentrations of methylprednisolone was observed. Therefore, the enhanced bactericidal effect of neutrophils by methylprednisolone cannot be explained by a direct bactericidal effect on the bacteria.

In general, an immunosuppressive treatment is described to affect the immune system by increasing susceptibility to infections [55]. Latent infections, presence of surgical transplants, airway compromise and skin diseases are risk factors for developing an infection when the application of immunosuppressive drugs is started [4,5]. Bacterial and fungal infections with manifestation at skin and urinary tract are common [56,57,58]. Side effects are mostly described for excessive and/or long-term treatment with immunosuppressive drugs [4,55,57,58]. Our detected bactericidal effect was observed for an early phase of infection due to the short incubation. Further investigations are needed to assess a long-term influence of used methylprednisolone concentrations on the bactericidal effect of neutrophils in vitro and in vivo afterwards. However, in good accordance with our findings, several studies describe immunosuppressive drugs associated with positive effects in infectious diseases [59,60,61]. For methylprednisolone, a suppressive effect on replication and survival of phagocytized bacteria in a concentration-dependent manner associated with the reduced production of IL-1β, IL-6 and TNF-α was demonstrated [59]. Another study suggests the inhibition of bacterial translocation from the gut and endotoxin release in rats with spinal cord injury after administration of high-dose methylprednisolone [60]. Furthermore, methylprednisolone has the property to protect the gastric mucosa from sepsis-induced gastric lesions, as shown in cats with *E. coli* bacteremia [61]. During simultaneous antibiotic treatment with moxalactam, neither positive or negative effect on bacterial clearance or on the kinetics of endotoxin release and clearance of induced *E. coli* sepsis in an experimental model after methylprednisolone administration could be detected [62].

Our results implicate that methylprednisolone enhances the bactericidal effect of canine neutrophils for infections with the Gram positive bacteria *St. pseudintermedius* and *Sc. canis*.

### 2.2. Methylprednislone Triggers the Release of Free DNA and Nucleosomes by Neutrophils during Bacterial Infection

Since we could show a concentration-dependent enhanced killing of Gram positive bacteria by neutrophils, we investigated the possible responsible mechanism. Bacteria can be killed by neutrophils with different mechanisms including phagocytosis, degranulation and the generation of ROS as well as the formation of NETs [13,15,16,17,18,63].

**Figure 2:**
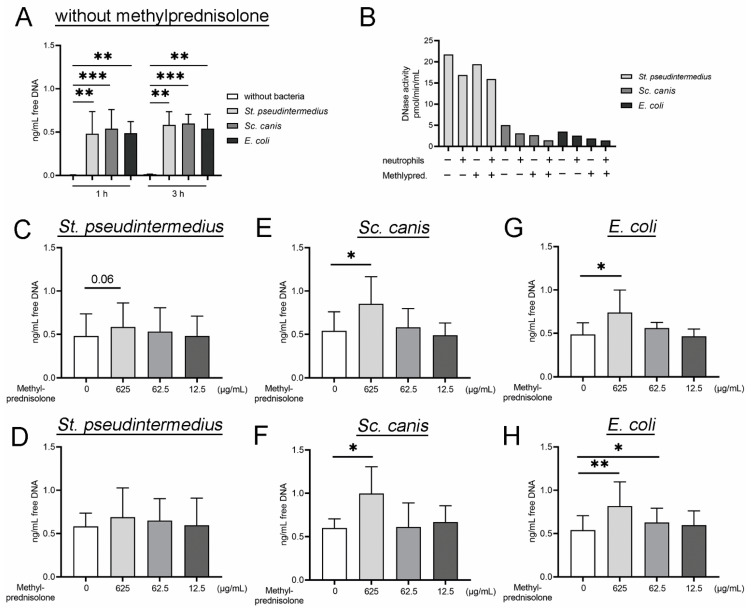
Free DNA is released by neutrophils after bacterial infection. The amount increased significantly in the presence of low DNase activity and methylprednisolone co-stimulation. The supernatants from bacterial killing assays from Figure 1 were analyzed for free DNA and partially for DNase activity. (**A**) Neutrophils incubated without methylprednisolone release free DNA into the supernatant after incubation with bacteria. *Sc. canis* induced the highest release of free DNA. (**B**) The DNase activity was determined in a selected sample collection (*n* = 1, 3 h post infection), including additional controls without neutrophils. Same tendencies in DNase activity were detected in all three bacterial strains. The highest DNase activity was detected in samples with *St. pseudintermedius*. In the presence of neutrophils, the DNase activity is slightly reduced. Furthermore, in the presence of 625 µg/mL methylprednisolone, the DNase activity is always reduced. (**C**,**D**) A notable higher amount of free DNA was detected only after 1 h of co-incubation from neutrophils with *St. pseudintermedius* and 625 µg/mL methylprednisolone. (**E**,**F**) A significant higher amount of free DNA was measured one and three hours after co-incubation of neutrophils with *Sc. canis* and 625 µg/mL methylprednisolone. (**E**,**F**) A significantly higher amount of free DNA was measured one and three hours after co-incubation of neutrophils with *E. coli* and 625 µg/mL and 62.5 µg/mL methylprednisolone, respectively. All graphs show the mean ± SD and statistical differences were detected by one-tailed paired Student’s *t*-test. The experiments were conducted in independent runs (1 h: *n* = 4; 3 h: *n* = 5). Data presented in A are shown partially again in (**C**–**H**) for statistical analysis in the different infection groups. Statistical results are presented for values lower than 0.1 and higher than 0.05 as Arabic numbers and as the following: *, *p* < 0.05; **, *p* < 0.01; ***, *p* < 0.001.

Free DNA release from neutrophils, as an indirect marker for NETs [19,25,28,64,65], was detected after neutrophils were incubated with the bacteria in the absence of methylprednisolone. The highest value was detected after incubation with *Sc. canis* (Figure 2A).

In presence of plasma neutrophils without methylprednisolone and with bacterial incubation released only a little amount of free DNA. Therefore, plasma does not seem to have a major impact on NET induction.

Nuclease activity (deoxyribonuclease (DNase)) was evaluated in the supernatants of the performed killing assay (Figure 2B), as DNases degrade NETs. All used bacterial species showed the same tendencies in nuclease activity, with the highest level detected for *St. pseudintermedius* and the lowest for *E. coli*. In the presence of neutrophils, the DNase activity is slightly reduced as well as in presence of 625 µg/mL methylprednisolone. DNase produced by *St. aureus* and *Sc. suis* represent an evasion mechanism for the microbes to degrade NETs and to protect them against antimicrobial activities of the NETs [66,67,68,69]. In addition to this mechanism, exogenous added DNase can contribute to an increase in phagocytosis shown for *Sc. pneumoniae* [70]. DNases are important for regulation of the amount of free DNA and, subsequently, of the NETs to maintain a balance by digestion of the DNA. After the digestion of the DNA, NETs lose antimicrobial activity [16]. An excess of NETs can result in dysfunction of the immune system and can result in organ dysfunction due to immunothrombosis [19] and is described in different diseases, especially of the immune system [18,19,71]. Our results indicate that neutrophils and methylprednisolone diminish bacterial DNase activity and therefore decreases the capacity of the bacteria to evade the NETs.

After co-incubation with methylprednisolone, *St. pseudintermedius* induced a notable higher release of free DNA from neutrophils only after one hour of incubation and only at a concentration of 625 µg/mL (Figure 2C,D). *Sc. canis* and *E. coli* induced a significant higher release of free DNA from neutrophils at different time points of incubation and methylprednisolone concentrations (Figure 2E–H). At a methylprednisolone concentration of 625 µg/mL, *Sc. canis* induced a significant higher release of free DNA from neutrophils at both time points (Figure 2E,F) compared to incubation of *Sc. canis* with neutrophils alone. After co-incubation of *E. coli* with neutrophils with and without methylprednisolone, statistically significant differences could be detected for 625 µg/mL methylprednisolone at one and three hours of incubation and for 62.5 µg/mL after three hours of incubation, respectively (Figure 2G,H). Based on the results we assumed that methylprednisolone results in concentration-dependent and time-dependent NET formation as an increased amount of DNA was detected. This could partially explain an enhanced bactericidal effect of neutrophils under methylprednisolone stimulation. Furthermore, in combination with the observed different DNase activity, it can be hypothesized that phagocytosis is partially influenced as well. However, further studies are needed to clarify this observation.

**Figure 3:**
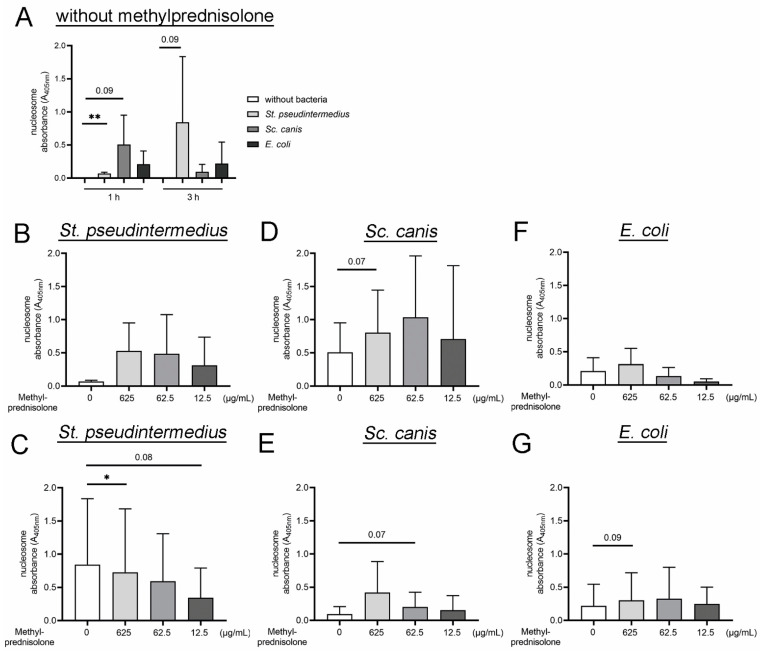
Nucleosomes are released by neutrophils after bacterial infection. The methylprednisolone co-stimulation influenced time and concentration dependent on the amount of nucleosomes in the supernatant. The supernatants from bacterial killing assays from Figure 1 were analyzed for nucleosomes. (**A**) Neutrophils incubated without methylprednisolone released nucleosomes into the supernatant after incubation with bacteria. *St. pseudintermedius* induced the highest release of nucleosomes after 1 h, whereas *Sc. canis* induced the highest release of nucleosomes after 3 h. (**B**,**C**) A higher amount of nucleosomes was detected after 1 h of co-incubation from neutrophils with *St. pseudintermedius* and methylprednisolone. After 3 h, the highest release was detected without co-incubation of methylprednisolone. (**D**,**E**) A notable higher amount of nucleosomes was measured one and three hours after co-incubation of neutrophils with *Sc. canis* and 625 µg/mL of methylprednisolone. (**F**,**G**) A tendency of higher amounts of nucleosomes was measured one and three hours after co-incubation of neutrophils with *E. coli* and 625 µg/mL of methylprednisolone. All graphs show the mean ± SD and statistical differences were detected by one-tailed paired Student’s *t*-test. The experiments were conducted in independent runs (1 h: *n* = 3; 3 h: *n* = 4). Data presented in A are shown partially again in (**B**–**G**) for statistical analysis in the different infection groups. Statistical results are presented for values lower than 0.1 and higher than 0.05 as Arabic numbers and as the following: *, *p* < 0.05; **, *p* < 0.01.

In order to analyze the released histones after bacterial infection and methylprednisolone incubation, histone-associated-DNA-fragments (mononucleosomes and oligonucleosomes) were quantified in the supernatants of treated neutrophils (Figure 3). In accordance with the detected levels of free DNA, nucleosome levels were significantly increased in the supernatants of infected neutrophils (Figure 3A)and indicate an additional NET-marker [72]. After one hour *St. pseudintermedius* induced the highest amount of nucleosomes, while after three hours *Sc. canis* was highly inducing the release of nucleosomes. However, in the presence of methylprednisolone, the release of nucleosomes by *St. pseudintermedius* was decreased in a concentration-dependent manner (Figure 3C). The release of nucleosomes after *Sc. canis* and *E. coli* infection was concentration-dependent and increased by methylprednisolone (Figure 3D–G). At three hours of incubation neutrophils released with all three bacteria always the highest nucleosome value with 625 µg/mL methylprednisolone incubation and the lowest value with 12.5 µg/mL methylprednisolone incubation. Interestingly, the release of nucleosomes changed between the two incubation time-points and is strain-dependent. While the release of nucleosomes was higher at one hour compared to three hours infection with *St. pseudintermedius*, the release of nucleosomes was lower at one hour compared to the three hour infection with *Sc. canis*. The amount of nucleosomes in the supernatant was comparable at one and three hours of infection with *E. coli* (Figure 3D–G). This observation shows that the reaction of neutrophils is influenced by the bacteria as well as by different concentrations of methylprednisolone.

Missing correlation of survival factors and the amount of free DNA within the single bacterial strains after co-incubation with neutrophils and methylprednisolone, especially for *St. pseudintermedius*, could be explained with the existence of nuclease activity produced by the bacterial species. *St. pseudintermedius* showed significant lower survival factors (Figure 1A,B) combined with the highest level of nuclease activity, which can result in the digestion of the NETs and subsequently lower amount of free DNA (Figure 2B–D). As the nucleosome detection is based on histones bound to DNA, this detection is influenced by the DNase activity as well. However, *St. pseudintermedius* showed the highest survival factor after three hours incubation with 12.5 µg/mL methylprednisolone (Figure 1B) and the lowest levels of nucleosome were detected in this experiment (Figure 3C).

Additionally, the detected quantitative amount of free DNA detected by Pico Green assay cannot distinguish NETs from double stranded DNA derived from other sources, for example, from release during apoptotic or necrotic cell death [19,73]. This can be an explanation for the high amount of free DNA release in the samples of *E. coli* (Figure 2G,H) and a lower amount of nucleosomes (Figure 3F,G), which lacks the corresponding significant lower survival factors (Figure 1E,F). The fact that *E. coli* as a Gram negative bacteria has lipopolysaccharides in the outer membrane which induces NET release can also result in the high amount of free DNA [19] to the suspected inductive property of methylprednisolone. A NET induction of *E. coli* was shown by immunofluorescence microscopy [74,75]. However, this NET release is not automatically associated with lower survival factors because *E. coli* is mainly killed intracellularly [76].

Furthermore, possible influences on NET release and digestion includes used autologous EDTA (sodium salt of ethylenediaminetetraacetic acid) plasma. For the plasma itself, a nuclease activity could not be ruled out completely and EDTA as a calcium and magnesium chelator could have an impact on NET release. Therefore, an assay without plasma was finally performed to confirm our assumed thesis that methylprednisolone triggers NET release.

### 2.3. Detection of NETs Induced by Methylprednisolone and Release of Free DNA and Nucleosomes in Absence of Plasma and Bacteria

**Figure 4:**
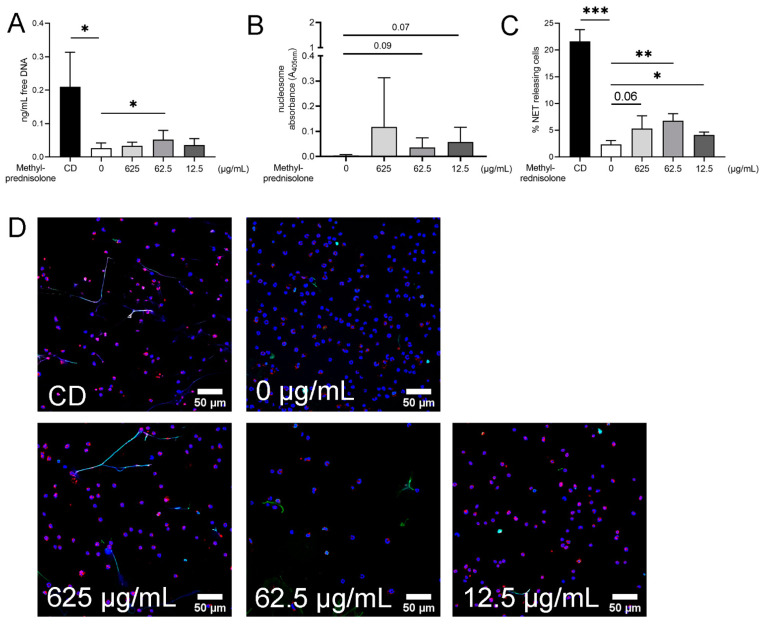
NETs are released from canine neutrophils after three hours of incubation with methylprednisolone in a concentration dependency. (**A**) Free DNA as one marker for NETs was detected in the supernatants of neutrophils incubated for three hours with different concentrations of methylprednisolone. Methyl-β-cyclodextrin (**C**,**D**) was used as a positive control. (**B**) Nucleosomes, as a further marker for NETs, were detected in the supernatants of neutrophils incubated for three hours with different concentrations of methylprednisolone. (**C**,**D**) By immunofluorescence microscopy, neutrophils were analyzed for NET release (green = DNA/histone-1 complexes, red = myeloperoxidase and blue = DNA). Per sample, six pictures were taken on two slides at predefined positions and the number of NET-releasing cells were determined by counting for statistical analysis. The settings were adjusted to a respective isotype control. Representative overlay pictures are presented in C. All graphs show the mean ± SD of *n* = 4 independent experiment and statistical differences were detected by one-tailed paired Student’s *t*-test. Statistical results are presented for values lower than 0.1 and higher than 0.05 as Arabic numbers and as the following: *, *p* < 0.05; **, *p* < 0.01; ***, *p* < 0.001. In C at 12.5 µg/mL, one outlier was excluded after analysis with Grubbs’ test.

In order to verify the hypothesis that methylprednisolone results in a release of free DNA from neutrophils, the quantification of free DNA in the supernatants by Pico Green Assay without bacteria and plasma was performed. Furthermore, nucleosomes were measured by ELISA and additionally immunofluorescence microscopy was conducted to quantify NETs. Immunofluorescence staining is one of the common methods for visualizing NETs in isolated neutrophil preparations, tissue sections, body fluid smears and in vivo. It is a more specific verification of NETs compared to analysis of free DNA alone; a combination of both methods is frequently used [19,28,75,77,78]. In the analysis of free DNA, a statistically higher amount was found at a concentration of 62.5 µg/mL methylprednisolone compared to the negative control of neutrophils alone (Figure 4A). Higher releases of nucleosomes by neutrophils were found under methylprednisolone treatment (Figure 4B). In the immunofluorescence microscopy, a significant increase in NET-releasing cells at a methylprednisolone concentration of 62.5 µg/mL could be detected. For a concentration of 625 µg/mL, a *p* value of 0.0603 was calculated. After Grubbs’ test an additional significance at a concentration of 12.5 µg/mL was noted (Figure 4C). Representative pictures of the immunofluorescence microscopy are presented in Figure 4D.

Next to the investigated effect of methylprednisolone on neutrophils in our study, other anti-inflammatory drugs are assessed regarding their influence on interaction with pathways of NET induction or even inhibition. Dexamethasone, also belonging to the group of glucocorticoids such as methylprednisolone, showed a NET inhibiting effect in patients with chronic inflammatory lung diseases after local application in contrast to methylprednisolone [79]. A decrease in NET formation in the lung of asthmatic horses after administration of dexamethasone could also be shown; however, in the blood samples, no differences were found in comparison to the controls [80]. Furthermore, dexamethasone could exacerbate fungal infections by a combination of reduced infiltration of neutrophils and inhibition of NET formationdescribed by Fan et al. [81]. In direct comparison, acetylsalicylic acid resulted in a marked suppression of NET formation along with increased bacteremia while dexamethasone had no evident effect on NET regulation [82]. In addition to the group of anti-inflammatory drugs, antibiotics can modulate the NET formation. Enrofloxacin enhances the formation of NETs in bovine granulocytes [34] and amoxicillin and clarithromycin could also be detected as a NET inducer [83,84]. In contrast gentamicin, azithromycin and chloramphenicol diminished NET release while clindamycin and cefotaxime had no influence on NET modulation [83,85,86]. Taken together, further studies are needed to understand the immunomodulating effects of different drugs and to identify new treatment approaches and risk factors during treatment of patients, especially since we observed the inhibiting and inducing effects in the presence of different methylprednisolone concentrations and different bacteria.

### 2.4. Other Neutrophil Mechanisms Influenced by Methlyprednisolone: Oxidative Burst, Phagocytosis, Chemotaxis and Cytokine Release

The recruitment of neutrophils is mediated via chemokines and follows a chemoattractant gradient towards the inflammatory site [87]. In the presence of inflammation, the chemokine receptor repertoire is changed and chemokine receptors for neutrophil phagocytic activity and ROS production are upregulated [88]. Due to the fact that ROS can be found in higher amounts when neutrophils are activated, we used ROS as a parameter for neutrophil activation. An increased amount of ROS can result in a powerful antimicrobial activity and in an increased transmigration or can enhance NET formation and NET mediated antimicrobial activity [89,90,91,92].

**Figure 5:**
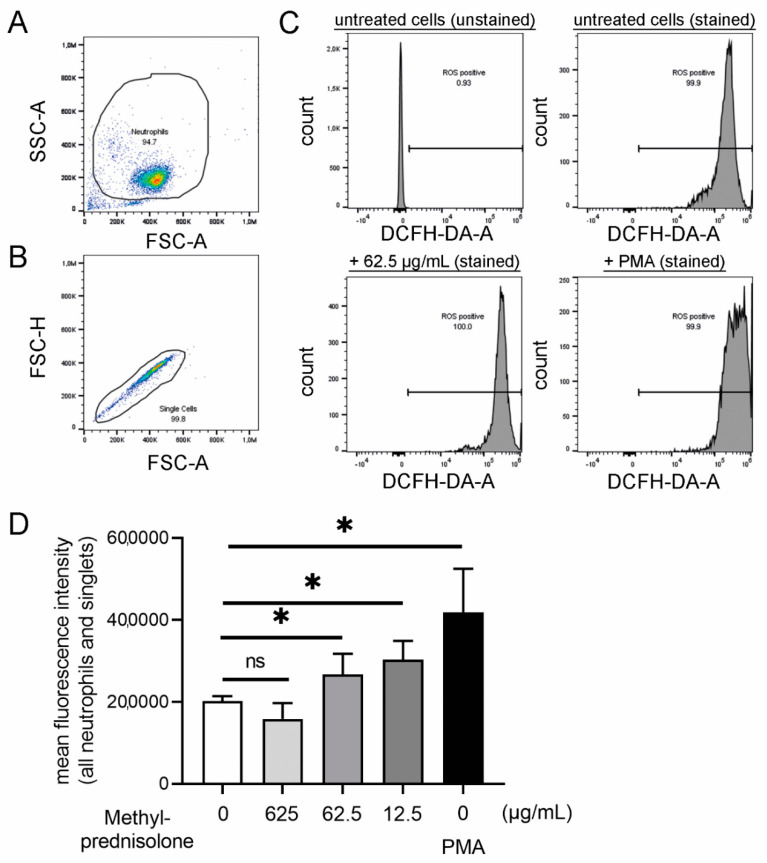
Isolated neutrophils produced concentration dependent higher amounts of intracellular reactive oxygen species (ROS). Phorbol 12-myristate 13-acetate (PMA) stimulation was used as the positive control in the absence of methylprednisolone. (**A**–**C**) The intracellular ROS production was determined by adding 2′7′ dichlorofluorescein diacetate (DCFH-DA) to unstimulated, methylprednisolone stimulated and PMA-stimulated cells. The gating strategy for the DCF-positive cells (oxidation of DCFH-DA by ROS results in fluorescence 2′7′ dichlorofluorosceindiacetate (DCF)) by flow cytometry is presented. (**A**) Based on FSC-A and SSC-A, the neutrophil population were gated. (**B**) Based on FSC-A and FSC-H, all singlets were gated from the neutrophil population. (**C**) Settings were adjusted in the gated population for ROS positive cells. Example histograms of different samples are presented. (**D**) The mean fluorescence intensity is presented and a concentration dependent increase in ROS positive cells was detectable after 45 min incubation. Data were analyzed with one-tailed paired Student’s *t*-test (*: *p* < 0.05) and are presented with mean ± SD (*n* = 3 experiments with the mean of duplicates are presented).

ROS levels increased in the presence of methylprednisolone in a concentration-dependent manner (Figure 5). At a concentration of 12.5 µg/mL methylprednisolone, the highest amount of ROS was produced by neutrophils. In the NET induction assay, the highest amount of NETs was observed with a methylprednisolone concentration of 62.5 µg/mL. However, these results can be explained by two points: (1) The ROS detection was performed after 45 min incubation with methylprednisolone whereas NETs were determined after three hours and (2) it can be hypothesized that methylprednisolone induces NETs not only via a ROS-dependent pathway as other pathways exist [93]. Further investigations are needed to determine the different ROS acting pathways, for example, by inhibiting the ROS-dependent NET release by adding diphenyleneiodonium (DPI) [91].

**Figure 6:**
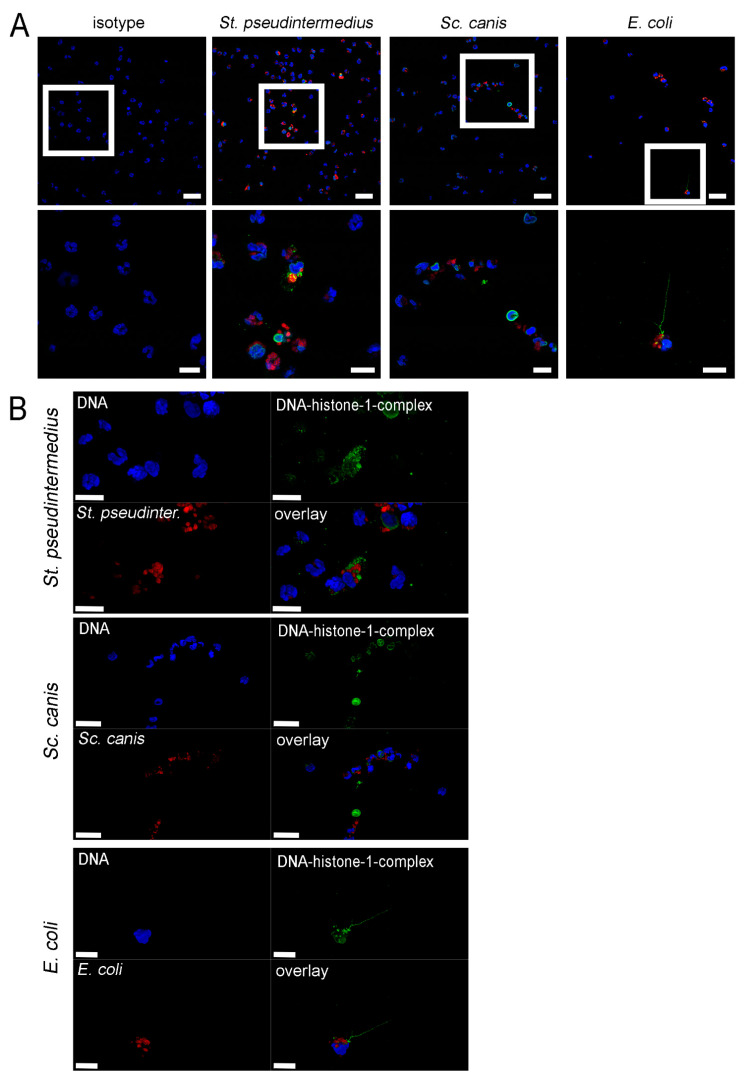
Phagocytosis and NET formation are detected in canine neutrophils after 3 h incubation with methylprednisolone. (**A**) By immunofluorescence microscopy, neutrophils were analyzed after three hours of incubation with 625 µg/mL methylprednisolone and bacteria for NET release and phagocytosed bacteria (Green = DNA/histone-1 complexes, red = bacteria and blue = DNA). For all three bacterial strains, bacteria were detected frequently close to the neutrophil nuclei and therefore these bacteria are probably at least partially phagocytosed. The settings were adjusted to a respective isotype control. Representative overlay pictures are presented. In the upper panel, the overview is presented and in the lower panel the zoom picture of the white square is presented. Scale bar upper panel = 25 µm, lower panel = 10 µm. (**B**) Representative 3D images of z-stacks from zoom pictures in A were constructed with LAS X 3D Version 3.1.0 software (Leica) (upper panel: 5.41 µm consisting of 44 sections, scale bar = 10 µm; middle panel: 5.54 µm consisting of 45 sections, scale bar = 20 µm; lower panel: 3.4 µm consisting of 28 sections, scale bar = 10 µm).

To confirm that methylprednisolone treated neutrophils can phagocytose and form NETs, further experiments with immunofluorescence microscopy analysis were performed. All three bacterial strains were observed in close distance to neutrophil nuclei and therefore a phagocytic activity of methylprednisolone treated neutrophils was possible (Figure 6). Furthermore, NET formation was detected. This observation results in the hypothesis that neutrophils undergo in response to bacteria and methylprednisolone vesicular NETosis and can therefore phagocytose bacteria in parallel. This phenomenon was described for bacteria such as *Staphylococcus aureus* and *E. coli* [93,94,95,96,97]. Further studies are needed to understand the mechanisms on how neutrophils release NETs in response to the bacteria in the presence of methylprednisolone, including electron microscopy analysis.

The effects of prednisolone on canine neutrophil functions were evaluated in different studies. After administration of prednisolone in healthy beagle dogs, the adherence of neutrophils decreased significantly while the chemotactic response to complement activation increased at some time points in vivo. Furthermore, the phagocytosis and killing capacity increased in vivo [98]. Controversially, methylprednisolone is able to suppress oxidative burst of neutrophils as it was demonstrated in healthy beagle dogs [99]. Similar results were found for hydrocortisone, which is another glucocorticoid [100]. Next to neutrophils, an inhibition of intracellular ROS production after prednisolone and dexamethasone administration is also described for platelets [101]. However, in osteocyte-like cells, dexamethasone increased the ROS level compared to control groups [102]. These results underline the findings of the present study as prednisolone seemed to support pathogen killing, NET release and ROS production in certain dosages. Consequently, different dosages of prednisolone may have varying effects on inflammatory response. The use of prednisolone in humans with experimentally induced endotoxemia resulted in a dose-dependent inhibition of cytokines (TNF α, IL-6) and chemokines (IL-8 and MCP-1), while anti-inflammatory cytokine concentrations increased [103]. Significant changes in cytokine expression were also shown in dogs, especially for TNF α, treated with either high doses of ciclosporin or a standard immunosuppressive dosage of prednisolone. TNF α concentrations significantly decreased [104]. This effect was also described in a canine endotoxemia model. Treatment with prednisolone attenuated IL-6 and TNF α concentrations dose-dependently [105]. Furthermore, the chemokine CCL17 was significantly increased in dogs with canine atopic dermatitis. Dogs treated successfully with prednisolone or oclacitinib showed a decrease in CCL17 but a direct effect of prednisolone on chemokine release remained debatable [106].

Our findings indicate that methylprednisolone serves as a potential NET inducer detected by immunofluorescence microscopy analysis and the determination of free DNA and nucleosomes. Furthermore, methylprednisolone influences the bactericidal effect of neutrophils on Gram positive bacteria during the early phase of co-incubation and influences dose-dependent intracellular ROS production.

## 3. Materials and Methods

### 3.1. Sample Collection

The collection of blood from healthy dogs was registered at the Lower Saxonian State Office for Consumer Protection and Food Safety (Niedersächsisches Landesamt für Verbraucherschutz und Lebensmittelsicherheit, No. 33.9-42502-05-18A250). It was conducted in line with the recommendations of the German Society for Laboratory Animal Science (Gesellschaft für Versuchstierkunde) and the German Veterinary Association for the Protection of Animals (Tierärztliche Vereinigung für Tierschutz e. V.) (GV-SOLAS Gesellschaft für Versuchstierkunde. Available online: http://www.gv-solas.de) (accessed on 27 April 2021). All blood taking procedures were conducted with oral consent from the dog owners.

In total, 10 adult clinical healthy client-owned dogs were included in this study. The study population consisted of eight males (Magyar Viszla (2), Golden Retriever, Malinois, Mixed breed dog (2), German wirehaired pointer and White shepherd dog) and two females (Mixed breed dogs). The maximum age for passing the inclusion criteria was defined at 8 years. From each dog, 10–13 mL of blood from the *Vena saphena lateralis* was taken and collected with anticoagulant EDTA. First, a blood count was performed from each blood sample by the Advia^®^120 System (Siemens Healthineers, Erlangen, Germany) to exclude white blood cell abnormalities (See Table A1).

### 3.2. Isolation of Canine Granulocytes

Purification of canine neutrophils were performed by density gradient centrifugation in combination with a hypotonic lysis of the erythrocytes. The density gradient was composed of 2 mL Histopaque 1.119 g/mL (Sigma-Aldrich, Germany) and 2 mL human Pancoll 1.077 g/mL (Pan Biotech GmbH, Aidenbach, Germany). The EDTA blood was diluted 1:2 with 1 x endotoxin-free PBS (phosphate buffered saline) (Sigma-Aldrich, Merck KGaA, Darmstadt, Germany). The amount of 6.5 mL blood-PBS mixture was layered on top of the gradient in a 15 mL falcon tube and the gradient was centrifuged (30 min, 700× *g* without brake, 20 °C). After removing plasma, peripheral blood mononuclear cells and Pancoll, the polymorphonuclear cells were harvested and diluted in 1 × endotoxin-free PBS. After centrifugation (10 min, 170× *g* with medium deceleration, 20 °C), the pelleted granulocytes were lysed for three times until the pellet was white. The hypotonic lysis was started after adding 6 mL sterile ice cold 0.2% sodium chloride (NaCl) to the pellet and stopped after 30 s by adding 6 mL of sterile ice cold 1.6% NaCl. Each lysis step was followed by a centrifugation (7 min, 280× *g* without brake at 4 °C). The purified pellet was resuspended in 1 mL Roswell Park Memorial Institute (RPMI) 1640 Medium (Thermo Fisher, 11,835,063 Gibco™ RPMI 1640 Medium, no phenol red, Carlsbad, CA, USA) and the number of polymorphonuclear cells was determined by counting the trypan blue (Merck KGaA, Darmstadt, Germany) negative cells using a Neubauer chamber.

The purity of the isolated canine granulocyte population was assessed by flow cytometry. Therefore, 1 mL propidium iodide (Thermo Fisher Scientific GmbH; Bremen, Germany) was diluted in 49 mL cell staining buffer (BioLegend, Sand Diego, CA, USA). For analysis by fluorescence-activating cell sorting (FACS), 20 µL of the cell suspension was mixed with 180 µL of the propidium iodide solution. The measurement was performed by using a MACSQuant^®^Analyzer 10 (Miltenyi Biotec B.V. & Co. KG, Bergisch Gladbach, Germany) while applying the Software MACSQuantify Software, (Miltenyi Biotec B.V. & Co. KG, Bergisch Gladbach, Germany). Ten thousand live-gated events were counted and the population of the granulocytes were detected by analysis of the cell size and granularity of the sample by forward scatter (FSC) and side scatter (SSC). On average, a purity of 90.5 ± 0.6% in the FACS analysis was achieved.

### 3.3. Bacterial Strains and Growth Conditions

In this study the following bacterial strains were used: *Streptococcus* (*Sc*.) *canis* (IMT48617), *Staphylococcus* (*St.*) *pseudintermedius* (IMT49419) and *Escherichia* (*E*.) *coli* (IMT45929). All strains were isolated from the wounds of dogs during diagnostic procedures at the Centre for Infection Medicine, Institute of Microbiology and Epizootics, Freie Universität Berlin, Germany. Bacterial strains were grown on a blood agar plate (Columbia Agar with 7% sheep blood; Thermo Scientific TM PB5008A, Waltham, MA, USA) at 37 °C (20–24 h).

Working cryostocks were produced as the following: One colony of *St. pseudintermedius* or *E. coli* was inoculated in 10 mL tryptic soy broth (TSB) without dextrose (Becton Dickinson, 286220, Franklin Lakes, NJ, USA) and incubated overnight at 37 °C, 200 rpm. A 1:50 dilution from the overnight culture was conducted with pre-warmed TSB (total 50 mL) and incubated at 37 °C 200 rpm until it reached the late exponential growth phase (*St. pseudintermedius*: OD_600nm_ 0.85 ± 0.05, *E. coli*: OD_600nm_ 2.1 ± 0.05).

Three colonies of *Sc. canis* were inoculated in 10 mL Todd Hewitt Broth (THB) (BD Bacto™ Dehydrated Culture Media: Todd Hewitt Broth; Becton Dickinson, 249240, Franklin Lakes, NJ, USA) and incubated at 37 °C 5% CO_2_ for around 16 h in a melting ice-bath to delay the start of growth. A 1:50 dilution from the overnight culture was conducted in pre-warmed THB (total 50 mL) and incubated at 37 °C, 5% CO_2_ until the late exponential growth phase (OD_600nm_ 0.9 ± 0.05).

Immediately, after the cultures reached the respective optical density, they were mixed with glycerol (final concentration of 15%) and aliquots in 1.5 mL tubes were shock frosted in liquid nitrogen. The working cryostocks were stored at −80 °C until they were used and only thawed once. The colony forming units (CFU/mL) were determined by the plating of serial dilutions on agar plates for each batch.

### 3.4. Neutrophil Killing Assay

The neutrophil killing assay was conducted as described previously [107] with the following changes. Canine neutrophils were seeded in 48 well plates (Greiner Bio-One, 677102, Kremsmünster, Austria) (2 × 10^5^ cells in a final volume of 200 µL/well) and infected with the three different bacterial strains from the working cryostocks (multiplicity of infection (MOI) = 1). Methylprednisolone (Solupred^®^ 62.5 mg/mL; cp Pharma, Burgdorf, Germany) in three different concentrations 625 µg/mL, 62.5 µg/mL and 12.5 µg/mL were additionally added. In the untreated sample (0 mg/mL), RPMI was added. Samples were incubated with 10% of autologous canine plasma. The plates were centrifuged (370× *g*, 5 min) and incubated afterwards for 1 and 3 h at 37 °C 5% CO_2_, respectively. After incubation, serial dilutions were conducted and plated on blood agar plates (*St. pseudintermedius* and *Sc. canis*) or on luria broth (LB) agar plates (*E. coli*). Plates were incubated for 20 h at 37 °C and the CFU/mL was determined for 0, 1 and 3 h. Finally, the survival factor was calculated as described previously [107] for each time-point.

Furthermore, at the end of the neutrophil killing assays, the 48 well plates were centrifuged (5 min, 370× *g* at 20 °C) and the supernatant was collected and stored at −20 °C for later analysis of free DNA (see below).

### 3.5. Survival of Bacteria in Presence of Methylprednisolone and Canine Plasma

The above-described neutrophil killing assay was conducted without neutrophils. The plates were incubated for 1 and 3 h at 37 °C 5% CO_2_. After incubation, serial dilutions were conducted and plated on blood agar plates (*St. pseudintermedius* and *Sc. canis*) or on luria broth (LB) agar plates (*E. coli*). Plates were incubated for 20 h at 37 °C and the CFU/mL was determined for 1 and 3 h.

### 3.6. NET Induction with Isolated Neutrophils

For the NET induction, assay cover slips (8 mm; Thermo Fisher Scientific GmbH, Bremen, Germany) were placed in 48 well plates (Greiner Bio-One, 677102, Kremsmünster, Austria). The slides were coated in accordance with the manufacturer’s instructions with poly-L-lysine (0.01% solution P4707, Sigma Aldrich, Munich, Germany) and handled afterwards, as previously described [108]. Harvested canine neutrophils were seeded in each well (2 × 10^5^/well). The final volume was 200 µL in each well. The cells were stimulated with methylprednisolone (Solupred^®^ 62.5 mg/mL; cp Pharma, Burgdorf, Germany) in three different concentrations, as described above. Negative control RPMI medium was added. A positive control methyl-β-cyclodextrin (CD, final concentration 10 mM; C4555 Sigma Aldrich, Munich, Germany) was added. After centrifugation (5 min, 370× *g*, 20 °C), the plates were incubated for 3 h at 37 °C and 5% CO_2_. Finally, cells were fixed with paraformaldehyde (4% final concentration). The plates were wrapped with parafilm and stored at 4 °C until the immunofluorescence staining was performed.

For the analysis of free DNA, cells and stimuli were co-incubated in 1.5 mL tubes in the same amount and concentrations as described for the 48 well plate experiment. Half-closed tubes were incubated for 3 h at 37 °C and 5% CO_2_. After centrifugation for 5 min, 400× *g* at 20 °C the supernatants were collected and stored at −20 °C for later analysis with a Pico Green assay (see below).

### 3.7. Immunofluorescence Staining of NETs

NETs were stained as previously described [32,107]. Briefly, after permeabilization and blocking, the samples were incubated with primary antibodies: A mouse monoclonal-antibody against DNA/histone 1 (1:1000 diluted in blocking buffer; Millipore MAB3864; 0.55 mg/mL, Billerica, MA, USA) and a rabbit polyclonal-antibody against human myeloperoxidase (1:337.5 diluted in blocking buffer; A039829-2 Agilent, Santa Clara, CA, USA, 3.2 mg) for 1 h. For the isotype controls, murine IgG2a (from murine myeloma, M5409-0.2 mg/mL, 1:364 Sigma Aldrich, Munich, Germany) and rabbit IgG (from rabbit serum, Sigma Aldrich, Munich, Germany, I5006, 1.16 mg, 1:108.75) were used in the blocking buffer. As the secondary antibody, a goat anti-mouse Alexa 488Plus-conjugated antibody (1:500 in blocking buffer, Invitrogen, Carlsbad, CA, USA) and a goat anti-rabbit Alexa 633-conjugated antibody (1:500 in blocking buffer; Thermo Fisher Scientific 2 mg, Waltham, MA, USA) were used for 1 h. Then, staining with aqueous Hoechst 33,342 (1:1000, stock 50 mg/mL, Sigma Aldrich, Munich, Germany) for 10 min was performed, while finally embedding the coverslips in ProLong^®^Gold antifade reagent (without DAPI, Invitrogen, Carlsbad, CA, USA).

### 3.8. Co-Staining of Bacteria and NETs

For visualization of bacteria and NETs by immunofluorescence microscopy, 48 well plates and coverslips were prepared as described above in the NET induction assay. The samples were prepared with neutrophils, 10% autologous plasma and bacteria as described above in the neutrophil killing assay and co-incubated with methylprednisolone (625 µg/mL). Afterwards, centrifugation samples were incubated for 3 h (37 °C, 5% CO_2_) and finally fixed with 4% paraformaldehyde. NETs were stained with a mouse monoclonal-antibody against DNA/histone 1 followed by an incubation with an ALEXA Flur488 Plus, as described above. *Sc. canis* was stained with a rabbit *anti-Streptococcus suis* antibody (self-made, 1:500 [109,110]). *St. pseudintermedius* was stained with a rabbit anti-*Staphylococcus aureus* antibody (IgG: stock 4 mg/mL, Abcam ab20920, 1:100). *E. coli* was stained with a rabbit anti *E. coli* (IgG: stock 4.5 mg/mL, novus biologicals, NB 200-579; 1:100). As the secondary antibody, all bacteria were stained with a goat anti-rabbit Alexa 633-conjugated antibody (1:500; Thermo Fisher Scientific 2 mg, Waltham, MA, USA) for 1 h. The staining was finally conducted as described above. Respective isotype controls were used as described above.

### 3.9. Immunofluorescence Microscopy and NET Quantification

Stained coverslips were recorded using a confocal inverted-base fluorescence microscope (Leica TCS SP5) with a HCX PL APO 40 × 0.75–1.25 oil immersion objective. Settings were adjusted with control preparations using an isotype control antibody. For each sample 6 randomly selected images were acquired and used for quantification. The cells present in the pictures were counted manually using the ImageJ software (version 1.52q, National Institute of Health, USA). The total number of neutrophils and positive neutrophils (activated or NET-releasing) were counted. A neutrophil was counted as positive if an evident off-shoot of DNA was visible or if at least two of the following criteria were found: enlarged nucleus, decondensed nucleus or blurry rim. The percentage of NET-positive neutrophils was calculated. For each sample, a minimum of 200 cells was counted and an average from the six pictures was calculated.

### 3.10. Pico Green Assay to Detect Free DNA

For detection of free DNA released from neutrophils without methylprednisolone stimulation, neutrophils were incubated as described in the neutrophil killing assays for 1 and 3 h. All samples were centrifuged and incubated and the supernatants were harvested after final centrifugation and stored at −20 °C for later analysis.

All collected samples from all assays were carefully defrosted and free DNA was quantified with a Pico Green Assay (Quant-iT^TM^ PicoGreen^®^dsDNA kit; Invitrogen, Carlsbad, CA, USA) following the manufacturer’s recommendation as previously described [107].

### 3.11. DNase Activity Assay

The Dnase activity assay Kit (BioVision, Milpitas, CA, USA, Fluorometric, K429-100) was used to determine the Dnase activity in the supernatant of the neutrophil killing assay and the respective controls. The test was performed following the manufacturer’s instructions with 25 µL for each sample.

### 3.12. Measurement of Nucloesome Fragments by ELISA

The cell death detection ELISA PLUS kit (Roche Diagnostics GmbH, Mannheim, Germany) was used to quantify the number of histone-associated-DNA-fragments (mono- and oligo-nucleosomes) in the supernatant of the neutrophil killing assay and the respective controls. The assay was conducted following the manufacturer’s instructions.

### 3.13. ROS Analysis

Intracellular ROS production was measured as described previously with small modifications [107]. Briefly, isolated neutrophils were incubated with methylprednisolone in three different concentrations (625 µg/mL, 62.5 µg/mL and 12.5 µg/mL) in 1.5 mL tubes. Stimulation with phorbol-myristate-acetate (PMA; Sigma-Aldrich, Munich, Germany) in a final concentration of 25 nM served as positive and unstimulated cells as negative control. Tubes were incubated for 15 min at 37 °C and 5% CO_2_. Subsequently 2′,7′-dichlorofluorescin-diacetate (DCFH-DA; Sigma Aldrich, Munich, Germany) with a final concentration of 10 µM was added to each sample and all samples were incubated at 37 °C and 5% CO_2_ for another 30 min. All samples were analyzed in duplicates and a respective background control without DCFH-DA was included. Intracellular ROS production was measured by flow cytometry (Attune^®^ NxT Acoustic Focusing Flow Cytometer, Invitrogen; Laser 488 nm (50 mW), filter BL1 = 530/30) and a total of 10,000 events were recorded. The mean green fluorescence intensity of all cells (X-Mean of BL-1) was determined as relative measurement of ROS production. The gating strategy included only singlets of the neutrophil population (See Figure 6). Data were analyzed with FlowJoTM10.7.1 software (Ashland, OR, USA).

### 3.14. Statistical Analysis

Data were analyzed using Excel (Microsoft Office) and GraphPad Prism 8.4.3 and 9.1.0 (GraphPad Prism Software, San Diego, CA, USA). For detection of statistical significances between two groups, an one-tailed paired Student’s *t*-test was performed. Probabilities lower than 0.05 were considered as statistically significant (*, *p* < 0.05, **, *p* < 0.01, ***, *p* < 0.001 and ****, *p* < 0.0001). Values higher than 0.05 and lower than or equal to 0.1 are presented as Arabic numbers. All values were analysed for normality with the Shapiro–Wilk test. Very few individual values did not pass the normality test, however, the statistical calculation was conducted for all values in all experiements equal.

## 4. Conclusions

In conclusion, methylprednisolone induces NET formation and intracellular ROS production in vitro. A concentration-dependent and time-dependent enhanced bactericidal effect of canine neutrophils was identified under methylprednisolone stimulation for the Gram positive *Staphylococcus pseudintermedius* and *Streptococcus canis*.

The clinical impact of these findings must be interpreted carefully. Recently, the use of low dose glucocorticoids (dexamethasone) gained attention as the mortality rate of hospitalized patients with COVID-19 who needed respiratory support was reduced [111]. In the early phase of bacterial infection, methylprednisolone might have a dose-dependent supportive effect regarding the immune defense of the host. Further studies are needed to investigate the in vivo influence of short-term methylprednisolone application on immune cells and infections as well as to investigate the long-term application effect.

## Abbreviations

CCL17	Thymus and activation-regulated chemokine
CFU	Colony forming unit
DNA	Deoxyribonucleic acid
DPI	Diphenyleneiodonium
*E. coli*	*Escherichia coli*
FACS	Fluorescence-activating cell sorting
h	Hours
IL-1	Interleukin 1
IL-6	Interleukin 6
IL-8	Interleukin 8
MCP-1	Monocyte chemoattractant protein-1
MOI	Multiplicity of infection
NET	Neutrophil extracellular trap
PMN	Polymorphonuclear granulocyte
ROS	Reactive oxygen species
RPMI	Roswell Park Memorial Institute
*Sc.*	*Streptococcus*
SD	Standard deviation
SF	Survival factor
*St.*	*Staphylococcus*
THB	Todd Hewitt Broth
TNF-α	Tumor necrosis factor alpha
TSB	Tryptic soy broth

## Appendix A

**Table A1:** Blood cell count of the included dogs.

Dog	WBC ×10^3^/µL (6–12)	PMNs ×10^3^/µL (3–10)	RBC ×10^6^/µL (6–9)	PLT ×10^3^/µL (150–500)	Blood Sample mL	Isolated PMNs ×10^6^/mL
1	7.91	5.02	6.23	226	10	19.6
2	10.97	7.26	6.66	221	13	24.6
3	6.27	3.81	6.16	232	10	14.9
4	10.63	6.73	7.4	204	10	19.9
5	6.38	3.23	7.23	199	10	17.1
6	8.11	5.62	7.43	358	12	26.1
7	6.92	4.45	6.19	169	11	13
8	5.43	2.81	6.68	206	12	15.6
9	8.87	5.59	7.08	242	10	16.6
10	7.24	4.43	6.9	122	13	26.8

WBC = white blood cell count; PMN = polymorphonuclear cells; RBC = red blood cell count; PLT = platelets.
